# Improved Speech Spatial Covariance Matrix Estimation for Online Multi-Microphone Speech Enhancement

**DOI:** 10.3390/s23010111

**Published:** 2022-12-22

**Authors:** Minseung Kim, Sein Cheong, Hyungchan Song, Jong Won Shin

**Affiliations:** School of Electrical Engineering and Computer Science, Gwangju Institute of Science and Technology, Buk-gu, Gwangju 61005, Republic of Korea

**Keywords:** multi-microphone speech enhancement, speech spatial covariance matrix estimation, temporal cepstrum smoothing, speech PSD estimation, RTF estimation

## Abstract

Online multi-microphone speech enhancement aims to extract target speech from multiple noisy inputs by exploiting the spatial information as well as the spectro-temporal characteristics with low latency. Acoustic parameters such as the acoustic transfer function and speech and noise spatial covariance matrices (SCMs) should be estimated in a causal manner to enable the online estimation of the clean speech spectra. In this paper, we propose an improved estimator for the speech SCM, which can be parameterized with the speech power spectral density (PSD) and relative transfer function (RTF). Specifically, we adopt the temporal cepstrum smoothing (TCS) scheme to estimate the speech PSD, which is conventionally estimated with temporal smoothing. Furthermore, we propose a novel RTF estimator based on a time difference of arrival (TDoA) estimate obtained by the cross-correlation method. Furthermore, we propose refining the initial estimate of speech SCM by utilizing the estimates for the clean speech spectrum and clean speech power spectrum. The proposed approach showed superior performance in terms of the perceptual evaluation of speech quality (PESQ) scores, extended short-time objective intelligibility (eSTOI), and scale-invariant signal-to-distortion ratio (SISDR) in our experiments on the CHiME-4 database.

## 1. Introduction

Speech enhancement is essential to ensure the satisfactory perceptual quality and intelligibility of speech signals in many speech applications, such as hearing aids and speech communication with mobile phones and hands-free systems [1,2,3,4,5,6,7,8,9,10,11,12,13,14,15,16,17,18,19,20,21,22,23,24,25,26,27,28,29,30,31,32,33,34,35,36,37,38,39,40,41,42,43]. Currently, devices with multiple microphones are popular, which has enabled multi-microphone speech enhancement, exploiting spatial information as well as spectro-temporal characteristics of the input signals [6,7,8,9,10,11,12,13,14,15,16,17,18,19,20,21,22,23,24,25,26,27,28,29,30,31,32,33,34,35,36,37,38,39,40,41,42,43,44,45,46,47,48]. One of the most popular approaches to multi-microphone speech enhancement may be spatial filtering in the time–frequency domain, which aims to extract a target speech signal from multiple microphone signals contaminated by background noise and reverberation by suppressing sounds from directions other than the target direction [6,7,8,9,10,11].

There have been various types of spatial filters with different optimization criteria [6,7,8,9,10]. Among them, the minimum mean square error (MMSE) criterion for speech spectra estimation led to the multi-channel Wiener filter (MWF), which has shown decent performance [10,12,21,22]. It has been shown that the MWF solution can be decomposed into the concatenation of the minimum-variance distortionless-response (MVDR) beamformer and the single-channel postfilter [11,12]. Spatial filters often require the estimation of acoustic parameters such as the relative transfer function (RTF) between the microphones and the speech and noise spatial covariance matrices (SCMs), which should be estimated from the noisy observations.

For applications such as speech communication and hearing aids, time delays are crucial, and thus an online algorithm is required for multi-microphone speech enhancement. The work in [25] extended the single-channel minima controlled recursive averaging (MCRA) framework [49,50] for noise estimation in the multi-channel case by introducing the multi-channel speech presence probability (SPP) [24]. In [26], a coherence-to-diffusion ratio (CDR) based *a priori* SPP estimator under the expectation and maximization (EM) framework was proposed to improve the robustness in nonstationary noise scenarios. In [25,26], the speech SCM was estimated with the maximum likelihood (ML) approach, while the multi-channel decision-directed (DD) estimator was proposed in [29]. In [27], the recursive EM (REM) algorithm, which performs an iterative estimation for the latent variables and model parameters in the current frame, was exploited by defining the exponentially weighted log-likelihood of the data sequence. The speech SCM was decomposed into the speech power spectral density (PSD) and RTF under the rank-1 approximation, and these components were estimated by an ML approach using the EM algorithm in [27].

In this paper, we propose an improved speech SCM estimation for online multi-microphone speech enhancement. First, we adopt the temporal cepstrum smoothing (TCS) approach [51] to estimate the speech PSD, which has not yet been tried in multi-channel cases. Furthermore, we propose an RTF estimator based on time difference of arrival (TDoA) estimation using the cross-correlation method. Finally, we propose refining the acoustic parameters by exploiting the clean speech spectrum and clean speech power spectrum estimated in the first pass. The experimental results show that the proposed speech enhancement framework exhibited improved performance in terms of the perceptual evaluation of speech quality (PESQ) scores, extended short-time objective intelligibility (eSTOI), and scale-invariant signal to distortion ratio (SISDR) for the CHiME-4 database. Additionally, we performed an ablation study to understand how each sub-module contributed to the performance improvement.

The remainder of this paper is organized as follows. Section 2 briefly introduces the previous work on multi-microphone speech enhancement depending on various classes of approaches and then summarizes the main contributions of our proposal. Section 3 reviews the previous MMSE multi-channel speech enhancement approach and explains the conventional speech and noise SCM estimation. Section 4 presents the proposed speech SCM estimation based on the novel speech PSD and RTF estimators. Section 5 outlines the experimental results that demonstrate the superiority of the proposed method compared with the baseline in terms of speech quality and intelligibility. Finally, a conclusion is provided in Section 6.

## 2. Previous Work and Contributions

Recently, many approaches to multi-microphone speech enhancement have been proposed. In [33], the estimation of the speech PSD reduces to seek a unitary matrix and the square roots of PSDs based on the factorization of the speech SCM. The RTF estimate was recursively updated based on these estimates. They also proposed a desmoothing of the generalized eigenvalues to maintain the non-stationarities of estimated PSDs. Furthermore, these parameter estimates were then exploited for a Kalman filter-based speech separation algorithm [35]. In the context of sound field analysis, ref. [34] proposed a masking scheme under the non-negative tensor factorization model and [36] exploited the sparse representation in a spherical harmonic domain. The work in [37] proposed a multi-channel non-negative factorization algorithm in the ray space transform domain.

Deep-learning-based approaches have also been proposed, which can be categorized into several types. One is the combination of deep learning with conventional beamforming methods, in which the deep neural networks (DNNs) are employed to implement beamforming [38,39]. In [38], the complex spectral mapping approach was proposed to estimate the speech and noise SCMs. In contrast, ref. [39] reformulated the MVDR beamformer as a factorized form associated with two complex components and estimated them using a DNN, instead of estimating the parameters of the MVDR beamformer. The other approach is neural beamforming, in which a DNN directly learns the relationship between multiple noisy inputs and outputs in an end-to-end way [40,41,42,43]. In [40], they defined spatial regions and proposed a non-linear filter that suppresses signals from the undesired region while preserving signals from the desired region. In [41], the authors proposed an end-to-end system to estimate the time-domain filter-and-sum beamformer coefficient using a DNN. This approach was later replaced with implicit filtering in latent space [42]. In [43], they built a causal neural filter comprising modules for fixed beamforming, beam filtering, and residual refinement in the beamspace domain.

One of the popular approaches that adapt the spatial filter according to the dynamic acoustic condition is the informed filter, which is computed by utilizing the instantaneous acoustic parametric information [15,16,17,18]. Refs. [15,16] exploited the instantaneous direction of arrival (DoA) estimates to find the time-varying RTF used to construct the spatial filter, and [18] formulated a Bayesian framework under the DoA uncertainty. In [19], the eigenvector decomposition was applied to the estimated speech SCM to extract the steering vectors, which were used for the MVDR beamformer. The aforementioned approaches often adopted classical techniques such as ESPRIT [52] or MUSIC [53] for DoA estimation, which may be improved by incorporating more sophisticated sound localization [47,48].

Another set of studies focus on the estimation of the acoustic parameters. An EM algorithm [14] was employed to perform a joint estimation of the signals and acoustic parameters. While clean speech signals were obtained in the E-step, the PSDs of signals, RTF, and SCMs were estimated in the M-step. As the previous EM algorithm processed all of the signal samples at once, REM algorithms [27,28] overcame these issues by carrying out frame-wise iterative processing to handle online scenarios. For the speech PSD estimation, ref. [32] proposed an instantaneous PSD estimation method based on generalized principal components to preserve the non-stationarity of speech signals. For the RTF estimation, previous approaches mainly exploited the sample SCMs [46]. The covariance subtraction (CS) approaches [44,45] estimated the RTF by taking the normalized first column of the SCM obtained by the subtraction of the noisy speech SCM and noise SCM, assuming that the rank of the speech SCM was one. On the other hand, the covariance whitening (CW) approaches [30,54] normalized the dewhitened principal eigenvector of the whitened noisy input SCM to obtain the RTF.

In this paper, we propose an improved speech SCM estimation method for the online multi-microphone speech enhancement system based on the MVDR beamformer–Wiener filter factorization. The main contributions of our proposals are as follows:

1. A speech PSD estimator based on the TCS scheme to take the knowledge on the speech signal in the cepstral domain into account;
2. An RTF estimator based on the TDoA estimate to take advantage of the information from all frequency bins, especially when the signal-to-noise ratio (SNR) is low;
3. The refinement of the acoustic parameter estimates by exploiting the clean speech spectrum and clean speech power spectrum estimated in the first pass.

## 3. MMSE Multi-Microphone Speech Enhancement

### 3.1. Signal Model

Suppose that there is an array of *M* microphones in a noisy and reverberant room. Assuming that a single speech source and noises are additive, the observed microphone signals are given as

(1) $$\begin{array}{cc} y(l,k) & =g\left(l,k\right)S_{1}\left(l,k\right)+v\left(l,k\right)\\ & =s(l,k)+v(l,k) \end{array}$$

where $y\left(l,k\right)={\left[Y_{1}\left(l,k\right),Y_{2}\left(l,k\right),\ldots ,Y_{M}\left(l,k\right)\right]}^{T}$, $s\left(l,k\right)={\left[S_{1}\left(l,k\right),S_{2}\left(l,k\right),\ldots ,S_{M}\left(l,k\right)\right]}^{T}$, and $v\left(l,k\right)=[V_{1}\left(l,k\right),$$V_{2}\left(l,k\right),...,V_{M}{\left(l,k\right)]}^{T}$, in which $Y_{m}\left(l,k\right)$, $S_{m}\left(l,k\right)$, and $V_{m}\left(l,k\right)$ are the short-time Fourier transform (STFT) coefficients of the microphone signal, clean speech, and background noises, including reverberations at the *m*th microphone, respectively, and $g\left(l,k\right)={\left[1,g_{2}\left(l,k\right),\ldots ,g_{M}\left(l,k\right)\right]}^{T}$ is the RTF vector for the direct path from the desired speech source to the microphones. We assume that $S_{m}\left(l,k\right)$ and $V_{m}\left(l,k\right)$ are uncorrelated as in [16], although early reflections may disrupt this assumption. The SCM for the input signal y(l,k), $\Phi_{y}\left(l,k\right)$, is given by

(2) $$\begin{array}{cc} \Phi_{y}\left(l,k\right) & =E[y\left(l,k\right)y^{H}\left(l,k\right)]\\ & =\Phi_{s}\left(l,k\right)+\Phi_{v}\left(l,k\right), \end{array}$$

where E[·] denotes mathematical expectation, and $\Phi_{s}\left(l,k\right)=E\left[s\left(l,k\right)s^{H}\left(l,k\right)\right]$ and $\Phi_{v}\left(l,k\right)=E\left[v\left(l,k\right)v^{H}\left(l,k\right)\right]$ are the SCMs of s(l,k) and v(l,k), respectively.

### 3.2. MWF and MVDR–Wiener Filter Factorization

The objective of multi-microphone speech enhancement is to estimate clean speech $S_{1}\left(l,k\right)$ from the noisy observation y(l,k), and we assume that prior knowledge on the location of the source or RTF is not available. One of the popular approaches is the MWF, which is a linear MMSE estimator for clean speech $S_{1}\left(l,k\right)$, i.e.,

(3) $$\begin{array}{c} {\hat{S}}_{1}\left(l,k\right)=w_{mwf}^{H}\left(l,k\right)y\left(l,k\right), \end{array}$$

where $w_{mwf}\left(l,k\right)$ denotes the MWF described as [6]

(4) $$\begin{array}{c} w_{mwf}\left(l,k\right)=\frac{\Phi_{v}^{-1}\left(l,k\right)\Phi_{s}\left(l,k\right)}{1+\mathrm{tr}(\Phi_{v}^{-1}\left(l,k\right)\Phi_{s}\left(l,k\right))}e_{1}, \end{array}$$

where $e_{1}={\left[\begin{array}{cc} 1 & 0_{1\times M-1} \end{array}\right]}^{T}$, in which **0** is a zero vector, and tr[·] denotes the trace of a matrix. It is noted that only the noise and speech SCM, $\Phi_{v}\left(l,k\right)$ and $\Phi_{s}\left(l,k\right)$, need to be estimated to implement the MWF. Previous work often adopted the multi-channel MCRA approach for noise SCM estimation, whereas ML estimation was employed for speech SCM estimation [25,26].

The MWF can be decomposed into the MVDR beamformer, $w_{mvdr}$, and a single-channel Wiener postfilter, $w_{wiener}$, as [11,12]

(5) $$\begin{array}{c} w_{mwf}=w_{mvdr}\cdot w_{wiener}, \end{array}$$

which makes it possible to consider the spatial filtering depending on the RTF **g** and the energy-based postfiltering $w_{wiener}$ separately. Note that the frame and frequency indices are omitted for notational convenience.

Let the output of the MVDR beamformer be *Z*, i.e.,

(6) $$\begin{array}{c} Z=w_{mvdr}^{H}y, \end{array}$$

where the MVDR beamformer is given as

(7) $$\begin{array}{c} w_{mvdr}=\frac{\Phi_{v}^{-1}g}{g^{H}\Phi_{v}^{-1}g}. \end{array}$$

With the distortionless constraint of the MVDR beamformer, the beamformer output can be expressed as [27]

(8) $$\begin{array}{c} Z=S_{1}+O, \end{array}$$

where *O* is assumed to follow the Gaussian distribution with variance

(9) $$\begin{array}{c} \phi_{o}={\left(g^{H}\Phi_{v}^{-1}g\right)}^{-1}. \end{array}$$

The clean speech spectrum can be obtained by applying the single-channel Wiener filter to the beamformer output *Z*, as

(10) $$\begin{array}{cc} {\hat{S}}_{1} & =\frac{\phi_{s}}{\phi_{s}+\phi_{o}}\cdot Z, \end{array}$$

where $\phi_{s}=E[|S_{1}{|}^{2}]$ is the speech PSD at the first microphone.

**Figure 1:**
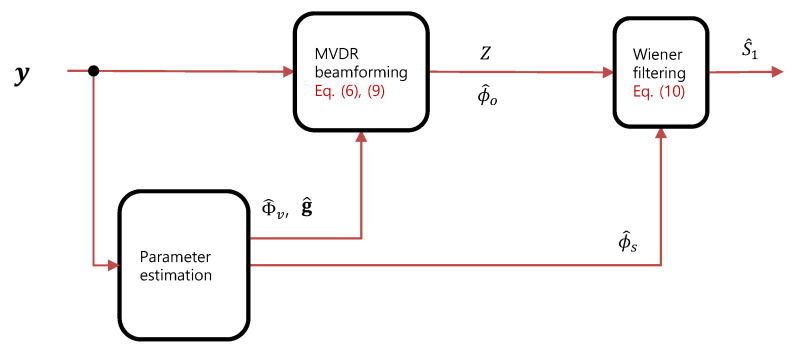
Block diagram of the multi-microphone speech enhancement system based on the MVDR–Wiener filter factorization.

Figure 1 illustrates the block diagram of the multi-microphone speech enhancement system based on the MVDR–Wiener filter factorization. The noisy speech *y* is processed by the MVDR beamformer and the Wiener filter sequentially, for which acoustic parameters $\Phi_{v}$, **g**, $\phi_{s}$, and $\phi_{o}$ need to be estimated. Existing methods for parameter estimation are present in the next subsection.

### 3.3. Speech and Noise SCM Estimation

As for the estimation of the SCM of noise, $\Phi_{v}$, the multi-channel MCRA approach [25] is widely used, which is given as

(11) $$\begin{array}{c} {\hat{\Phi }}_{v}\left(l,k\right)={\tilde{\alpha }}_{v}\left(l,k\right){\hat{\Phi }}_{v}\left(l-1,k\right)+\left(1-{\tilde{\alpha }}_{v}\left(l,k\right)\right)y\left(l,k\right)y^{H}\left(l,k\right), \end{array}$$

where ${\tilde{\alpha }}_{v}\left(l,k\right)=\lambda +p\left(H_{1}\left(l,k\right)|y\left(l,k\right)\right)\left(1-\lambda \right)$ is an SPP-dependent smoothing parameter with a constant 0<λ<1. This method updates $\Phi_{v}$ more when the SPP is low and vice versa. The *a posteriori* SPP $p(H_{1}|y)$ can be obtained using Bayes’ rule as

(12) $$\begin{array}{c} p\left(H_{1}|y\right)=\frac{p\left(H_{1}\right)p\left(y|H_{1}\right)}{p\left(H_{0}\right)p\left(y|H_{0}\right)+p\left(H_{1}\right)p\left(y|H_{1}\right)}, \end{array}$$

where $H_{0}$ and $H_{1}$ denote the hypotheses for speech absence and presence, respectively, and $p(y|H_{0})$ and $p(y|H_{1})$ are modeled as complex multivariate Gaussian distributions, as follows:

(13) $$\begin{array}{cc} p(y|H_{0}) & =\frac{1}{\pi^{M}\det\left[\Phi_{v}\right]}\exp-y^{H}\Phi_{v}^{-1}y \end{array}$$

(14) $$\begin{array}{cc} p(y|H_{1}) & =\frac{1}{\pi^{M}\det\left[\Phi_{v}+\Phi_{s}\right]}\exp\{-y^{H}{\left[\Phi_{v}+\Phi_{s}\right]}^{-1}y\}, \end{array}$$

in which det[·] denotes the determinant of a matrix. Then, $p(H_{1}|y)$ becomes [24]

(15) $$\begin{array}{c} p\left(H_{1}|y\right)={\left(1+\frac{p(H_{0})}{p(H_{1})}\left(1+\xi \right)\exp\{-\frac{\beta }{1+\xi }\}\right)}^{-1}, \end{array}$$

where $\xi =\mathrm{tr}(\Phi_{v}^{-1}\Phi_{s})$, $\beta =y^{H}\Phi_{v}^{-1}\Phi_{s}\Phi_{v}^{-1}y$, and $p\left(H_{1}\right)=1-p\left(H_{0}\right)$ is the *a priori* SPP, which can be estimated using the CDR-based [26] or DNN-based [27] method.

The speech SCM is usually estimated with the ML approach, which is defined as [25,26]

(16) $$\begin{array}{c} {\hat{\Phi }}_{s}^{ml}={\hat{\Phi }}_{y}-{\hat{\Phi }}_{v}, \end{array}$$

where ${\hat{\Phi }}_{y}$ is obtained by recursive smoothing as

(17) $$\begin{array}{c} {\hat{\Phi }}_{y}\left(l,k\right)=\lambda {\hat{\Phi }}_{y}\left(l-1,k\right)+\left(1-\lambda \right)y\left(l,k\right)y^{H}\left(l,k\right). \end{array}$$

Under the rank-1 approximation for the clean speech SCM, ${\hat{\Phi }}_{s}$ can be further refined using the decomposition of $\Phi_{s}$, with the speech PSD and RTF given by [8]

(18) $$\begin{array}{cc} \Phi_{s} & =\phi_{s}gg^{H}. \end{array}$$

Adopting the covariance subtraction (CS) approach, which extracts the normalized first column vector of the ML estimator of the speech SCM ${\hat{\Phi }}_{s}^{ml}$, the estimator for the RTF is given as [46]

(19) $${\hat{g}}^{cs}=\frac{{\hat{\Phi }}_{s}^{ml}e_{1}}{e_{1}^{T}{\hat{\Phi }}_{s}^{ml}e_{1}},$$

where the denominator represents the speech PSD, i.e.,

(20) $${\hat{\phi }}_{s}^{cs}=e_{1}^{T}{\hat{\Phi }}_{s}^{ml}e_{1},$$

in which the superscript ${}^{cs}$ indicates the CS approach.

In the REM framework [27], the ML estimator for the RTF based on the observed noisy speech is obtained as [27]

(21) $$\begin{array}{c} {\hat{g}}^{ml}\left(l,k\right)=\frac{\sum_{\tau =1}^{l}\lambda^{l-\tau }p\left(H_{1}\left(\tau ,k\right)|y\left(\tau ,k\right)\right)y\left(\tau ,k\right){\hat{S}}_{1}^{*}\left(\tau ,k\right)}{\sum_{\tau =1}^{l}\lambda^{l-\tau }p\left(H_{1}\left(\tau ,k\right)|y\left(\tau ,k\right)\right){\hat{S}}_{1}\left(\tau ,k\right){\hat{S}}_{1}^{*}\left(\tau ,k\right)}, \end{array}$$

where the summations in the numerator and the denominator can be computed with recursive averaging. The numerator can be thought of as the estimate of the cross-correlation between **y** and ${\hat{S}}_{1}$ in (10), ${\hat{r}}_{ys}$, given by

(22) $$\begin{array}{c} {\hat{r}}_{ys}\left(l,k\right)=\lambda {\hat{r}}_{ys}\left(l-1,k\right)+\left(1-\lambda \right)p\left(H_{1}\left(l,k\right)|y\left(l,k\right)\right)y\left(l,k\right){\hat{S}}_{1}^{*}\left(l,k\right), \end{array}$$

and the denominator can be considered to be the estimate of the speech PSD obtained by the recursive smoothing of the estimated clean speech power spectrum,

(23) $${\hat{\phi }}_{s}^{ts}\left(l,k\right)=\lambda {\hat{\phi }}_{s}^{ts}\left(l-1,k\right)+\left(1-\lambda \right)\hat{|S_{1}{|}^{2}}\left(l,k\right),$$

where the superscript ${}^{ts}$ indicates it is a temporally smoothed estimate, and $\hat{|S_{1}{|}^{2}}\left(l,k\right)$ is the MMSE estimator of $|S_{1}{|}^{2}$ under the speech presence uncertainty given by

(24) $$\begin{array}{cc} \hat{|S_{1}{|}^{2}} & =E[|S_{1}{|}^{2}|Z]\\ & =p\left(H_{1}|Z\right)\cdot E[|S_{1}{|}^{2}|Z,H_{1}]\\ & =p\left(H_{1}|y\right)\cdot ({\left(\frac{\phi_{s}}{\phi_{s}+\phi_{o}}\right)}^{2}\cdot {\left|Z\right|}^{2}+\frac{\phi_{s}\phi_{o}}{\phi_{s}+\phi_{o}}), \end{array}$$

where we let $p\left(H_{1}|Z\right)=p\left(H_{1}|y\right)$, as in [27]. With ${\hat{r}}_{ys}$ in (22) and ${\hat{\phi }}_{s}^{ts}$ in (23), ${\hat{g}}^{ml}$ in (21) can be expressed as

(25) $$\begin{array}{cc} {\hat{g}}^{ml} & =\frac{{\hat{r}}_{ys}}{{\hat{\phi }}_{s}^{ts}}. \end{array}$$

## 4. Proposed Speech SCM Estimation

**Figure 2:**
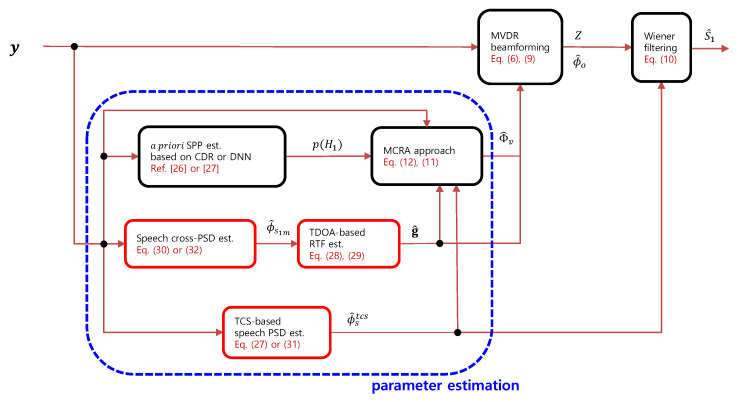
Block diagram of the proposed multi-microphone speech enhancement system.

Figure 2 illustrates the block diagram of the proposed speech enhancement system. As in [25,26,27], the estimation of the speech and relevant statistical parameters is performed twice for each frame, which was shown to be effective for online speech enhancement. In this paper, we propose an improved method for speech SCM estimation, i.e., speech PSD estimation and RTF estimation with a rank-1 approximation, using the speech enhancement system described in Figure 2. Note that the proposed modules are highlighted with red boxes.

In the first pass, we exploit the noisy input y(l) in the current frame and the noise SCM estimate ${\hat{\Phi }}_{v}\left(l-1\right)$ obtained in the previous frame to estimate the acoustic parameters in the current frame and perform beamforming and postfiltering, as explained in Section 3.2.

The ML estimate of the speech PSD at the first microphone using an instantaneous estimate of the PSD of input noisy signal can be obtained as

(26) $$\begin{array}{c} {\hat{\phi }}_{s}^{ml}\left(l,k\right)=\max\left(|Y_{1}\left(l,k\right){|}^{2}-{\hat{\phi }}_{v}\left(l-1,k\right),{\hat{\phi }}_{s}^{min}\right), \end{array}$$

where ${\hat{\phi }}_{v}\left(l-1,k\right)$ is the (1,1)th component of ${\hat{\Phi }}_{v}\left(l-1,k\right)$, and ${\hat{\phi }}_{s}^{min}$ is a certain minimum value for the speech PSD estimate, which is set as $\xi^{min}{\hat{\phi }}_{v}\left(l-1,k\right)$ with a tunable parameter $\xi^{min}$. To estimate the speech PSDs, the ML estimation with temporal smoothing has been commonly used as described in (16) and (17) [25,26,27]. However, this approach occasionally results in undesired temporal smearing of speech [51]. In this paper, we propose to apply TCS [51] to ${\hat{\phi }}_{s}^{ml}$ in (26). TCS is a selective temporal smoothing technique in the cepstral domain motivated by the observation that, although the excitation component resides in a limited number of cepstral coefficients dependent on the pitch frequency, the speech spectral envelope is well-represented by the cepstral bins with low indices [55]. Specifically, the TCS consists of the following procedure: First, the cepstrum of ML speech PSD estimate ${\hat{\phi }}_{s}^{ml,ceps}\left(l,q\right)$ is computed by the inverse discrete Fourier transform (IDFT) of ${\hat{\phi }}_{s}^{ml}$. Next, the selective smoothing is applied to ${\hat{\phi }}_{s}^{ml,ceps}\left(l,q\right)$, in which the cepstral bins that are less relevant to speech are smoothed more and those representing the spectral envelope and fundamental frequency are less smoothed. Finally, the discrete Fourier transform is used to convert ${\hat{\phi }}_{s}^{ceps}\left(l,q\right)$ into the TCS-based speech PSD estimate in the spectral domain ${\hat{\phi }}_{s}^{tcs}\left(l,k\right)$. The bias compensation for the reduced variance due to the cepstral smoothing can be found in [56], and a detailed description of the adaptation of the smoothing parameters and the fundamental frequency estimation is given in [51]. In this paper, we denote the aforementioned procedure of TCS as an operation:

(27) $$\begin{array}{cc} {\hat{\phi }}_{s}^{tcs,f}\left(l\right) & =\mathrm{TCS}({\hat{\phi }}_{s}^{ml}\left(l\right)), \end{array}$$

in which the superscript ${}^{f}$ indicates that this is the estimate in the first pass.

In this paper, we model the RTF vector *g* as a relative array propagation vector, which depends on the DoA [16]. Note that the conventional approaches in [27,44] estimate the RTF for each frequency using the input statistics in the frequency bin, ignoring the inter-frequency dependencies. In the presence of heavy noise, the accurate estimation of the RTF may become difficult, and thus it would be beneficial to estimate TDoA by utilizing the input signal in all frequency bins and to reconstruct the RTF using the simplest model. The TDoA for the desired speech can be obtained from the estimate of the cross-PSD of the desired speech, $\phi_{s_{1m}}\left(l,k\right)=E\left[S_{1}\left(l,k\right)S_{m}^{*}\left(l,k\right)\right]$, using the cross-correlation method [57]. The TDoA estimate $\tau_{m}$ between the first and the *m*th microphones is given by

(28) $$\begin{array}{cc} \tau_{m}\left(l\right) & =\arg\max_{\tau }\gamma_{1m}\left(\tau \right),\\ \gamma_{1m}\left(\tau \right) & ≜\sum_{k=1}^{K}{\hat{\phi }}_{s_{1m}}\left(l,k\right)\cdot e^{j2\pi k\tau /K}, \end{array}$$

in which ${\hat{\phi }}_{s_{1m}}\left(l,k\right)$ is the estimate of $\phi_{s_{1m}}\left(l,k\right)$. Then, the TDoA-based RTF estimator can be obtained as

(29) $${\hat{g}}^{tdoa}\left(l,k\right)=\exp\{j2\pi k\bar{\tau }\left(l\right)/K\},$$

where $\bar{\tau }={\left[\tau_{1},\tau_{2},...,\tau_{M}\right]}^{T}$.

In the first pass, the cross-PSD estimate ${\hat{\phi }}_{s_{1m}}$ can be obtained by taking the (1,m) element of the ML speech SCM estimate ${\hat{\Phi }}_{s}^{ml}\left(l,k\right)$ in (16) as

(30) $$\begin{array}{c} {\hat{\phi }}_{s_{1m}}^{f}\left(l,k\right)=e_{m}^{T}{\hat{\Phi }}_{s}^{ml}\left(l,k\right)e_{1}, \end{array}$$

where $e_{m}={\left[\begin{array}{ccc} 0_{\left(m-1\right)} & 1 & 0_{\left(M-m\right)} \end{array}\right]}^{T}$ in which $0_{n}$ is an all-zero vector of length *n*; ${\hat{g}}^{tdoa,f}$ can be computed using (28) and (29) with ${\hat{\phi }}_{s_{1m}}^{f}$, and ${\hat{\Phi }}_{s}^{f}$ can be obtained as in (18) using ${\hat{\phi }}_{s}^{tcs,f}\left(l\right)$ in (27) and ${\hat{g}}^{tdoa,f}$. The noise SCM is estimated with the multi-channel MCRA approach in (11) utilizing $p(H_{1}|y)$ in (15) computed with ${\hat{\Phi }}_{s}^{f}$ and ${\hat{\Phi }}_{v}$. Then, we can compute the beamformer output *Z* in (6) and $\phi_{o}$ in (9), and the estimate for the speech spectrum, ${\hat{S}}_{1}$, can be obtained as in (10).

In the second pass, we estimate the acoustic parameters again by additionally utilizing the estimates for the clean speech spectrum, clean speech power spectrum, and *a posteriori* SPP, computed in the first pass. These refined parameters are in turn used to estimate the clean speech once again.

To refine the estimate of the speech PSD, we apply the TCS to the clean speech power spectrum estimate $\hat{|S_{1}{|}^{2}}$ in (24) as

(31) $$\begin{array}{cc} {\hat{\phi }}_{s}^{tcs,r}\left(l\right) & =\mathrm{TCS}(\hat{|S_{1}{|}^{2}}\left(l\right)), \end{array}$$

in which the superscript ${}^{r}$ indicates it is the refined estimate in the second pass. As $\hat{|S_{1}{|}^{2}}$ would be less affected by the noise compared with the ${\hat{\phi }}_{s}^{ml}$ by virtue of beamforming and the MMSE estimation, ${\hat{\phi }}_{s}^{tcs,r}\left(l\right)$ would be more accurate than ${\hat{\phi }}_{s}^{tcs,f}\left(l\right)$. As for the RTF estimation, ${\hat{r}}_{ys}$ in (22) is evaluated with ${\hat{S}}_{1}$ in (10), as in [27]. Instead of using ${\hat{r}}_{ys}$ divided by the estimate of the speech PSD in the first microphone to obtain the RTF, as in [27], we again estimate the RTF based on the TDoA; ${\hat{\phi }}_{s_{1m}}$ can be computed by extracting the *m*th element of ${\hat{r}}_{ys}$ as

(32) $${\hat{\phi }}_{s_{1m}}^{r}\left(l,k\right)=e_{m}^{T}{\hat{r}}_{ys}\left(l,k\right),$$

in contrast to (30). The TDoA-based RTF estimate in the second pass, ${\hat{g}}^{tdoa,r}$, can be obtained through (28) and (29) with ${\hat{\phi }}_{s_{1m}}^{r}$. As in the first pass, ${\hat{\Phi }}_{s}^{r}$ is computed with ${\hat{\phi }}_{s}^{tcs,r}$ in (31) and ${\hat{g}}^{tdoa,r}$, and $p(H_{1}|y)$ in (15) is updated with ${\hat{\Phi }}_{s}^{r}$. Then, $p(H_{1}|y)$ and ${\hat{\Phi }}_{v}$ are obtained again using (15) and (11), and then the beamformer output *Z* and $\phi_{o}$ are updated using (6) in (9). The final clean speech estimate ${\hat{S}}_{1}$ is obtained by (10) using ${\hat{g}}^{tdoa,r}$, ${\hat{\Phi }}_{v}$, and ${\hat{\phi }}_{s}^{tcs,r}$. The whole procedure of the proposed online multi-microphone speech enhancement method is summarized in Algorithm 1.
**Algorithm 1** Proposed multi-microphone speech enhancement algorithm with improved speech SCM estimation. 1:**Inputs**: ***y*** for all frames 2:**Output**: ${\hat{S}}_{1}$ for all frames 3:**Initialize** variables and parameters 4:**for** each frame **do** 5:     Compute $p(H_{1})$ using CDR-based [26] or DNN-based [27] method 6:     **(First pass)** 7:     Compute ${\hat{\phi }}_{s}^{tcs,f}$ via (26) and (27) 8:     Compute ${\hat{g}}^{tdoa,f}$ via (16), (28)–(30) 9:     Estimate $p(H_{1}|y)$ and ${\hat{\Phi }}_{v}$ via (11), (15) and (18)10:   Beamformer: Compute *Z* and $\phi_{o}$ via (6) and (9)11:   Postfilter: Compute ${\hat{S}}_{1}$ via (10)12:   **(Second pass)**13:   Compute ${\hat{\phi }}_{s}^{tcs,r}$ via (24) and (31)14:   Compute ${\hat{g}}^{tdoa,r}$ via (22), (28), (29) and (32)15:   Estimate $p(H_{1}|y)$ and ${\hat{\Phi }}_{v}$ via (11), (15) and (18)16:   Beamformer: Compute *Z* and $\phi_{o}$ via (6) and (9)17:   Postfilter: Compute ${\hat{S}}_{1}$ via (10)18:**end for**

## 5. Experiments

### 5.1. Experimental Settings

To demonstrate the superiority of the proposed algorithm, we conducted a set of experiments to evaluate the performance of the multi-microphone speech enhancement on the simulated set in the CHiME-4 database [58]. In this database, a mobile tablet device with six microphones was used for recording, of which the three microphones numbered 1, 2, and 3 were located in the top left, center, and right with an inter-microphone distance of approximately 10 cm each, while the other three microphones numbered 4, 5, and 6 were placed in the bottom left, center, and right, respectively [58]. The vertical distance between pairs of microphones was approximately 19 cm [58]. All microphones were located on the frontal surface, except for microphone 2. The bus (BUS), cafe (CAF), pedestrian area (PED), and street junction (STR) types of noise were used, and the SNR was between 0 and 15 dB. The training set consisted of 7138 utterances spoken by 83 speakers, whereas the development and evaluation sets were 1640 utterances and 1320 utterances, respectively, from 4 different speakers. The sampling rate for the signals used in the experiments was 16 kHz, and the square-root Hann window was applied to a 32 ms signal with a 16 ms frame shift. The 512-point DFT was applied to the windowed signal. The reference channel for the algorithms and evaluations was microphone 5, located at the bottom center of the device.

We set the *a posteriori* SPP, $p(H_{1}|y)$, to zero for the first 10 frames instead of computing it using (15) based on the assumption that the speech would be absent in the initial periods, which helped the fast stabilization of the algorithm, as in [27]. To mitigate speech distortion at the expense of increased residual noise [59], the lower bounds for the $p(H_{1}|y)$ to compute $\hat{|S_{1}{|}^{2}}$ in (24) and the Wiener gain in (10) were configured to 0.5 and −18 dB, respectively. The parameter values for λ and $\xi^{min}$ were set to be 0.9 and −10 dB, respectively. For the TCS schemes in (27) and (31), we followed the procedure in [51], employing the same parameter values except for the constant smoothing parameter, ${\bar{\alpha }}^{\mathrm{const}}$, which was determined empirically as

(33) $$\begin{array}{cc} {\bar{\alpha }}^{\mathrm{const}}\left(q\right) & =\left\{\begin{array}{cc} 0.1 & \mathrm{if}\;q\in \{0,\ldots ,2\}\\ 0.5 & \mathrm{if}\;q\in \{3,\ldots ,19\}\\ 0.95 & \mathrm{if}\;q\in \{20,\ldots ,256\}, \end{array}\right. \end{array}$$

in which *q* is the quefrency index.

For the DNN-based *a priori* SPP estimation, we adopted the DNN architecture in [27], which consisted of a uni-directional long short-term memory (LSTM) layer of 512 dimensions, followed by three fully-connected layers of 256 dimensions. The activation functions were the rectified linear unit (ReLU) for the first three layers and sigmoidal activation for the last layer, which produced a 257-dimensional output vector. The number of dimensions of the DNN output is 257. The input for the DNN was the noisy log magnitude spectrum at the reference microphone, and the training target was binary for each bin, which was set by thresholding the instantaneous SNR [13].

### 5.2. Experimental Results

To demonstrate the superior performance of the proposed speech enhancement method, we evaluated the wideband PESQ score [60], eSTOI [61], and SISDR [62]. As we focused on the online framework in which the algorithm only uses the current and previous audio samples for frame-wise processing, the online algorithms designed in this way were chosen for the baseline methods. Depending on the *a priori* SPP estimator, we compared the performance of the proposed method using the ML framework with the MWF in [26] when the CDR-based *a priori* SPP estimator [26] was adopted, whereas the REM approach [27] was used for performance comparison when the DNN-based *a priori* SPP estimator [27] was employed for the proposed algorithm. As in [27], two versions of the REM approach using the Wiener postfilter and Kalman postfilter, denoted by DNN-REMWF and DNN-REMKF, were included in the experiment. The configuration parameters for the compared methods were set as in the original papers.

**Figure 3:**
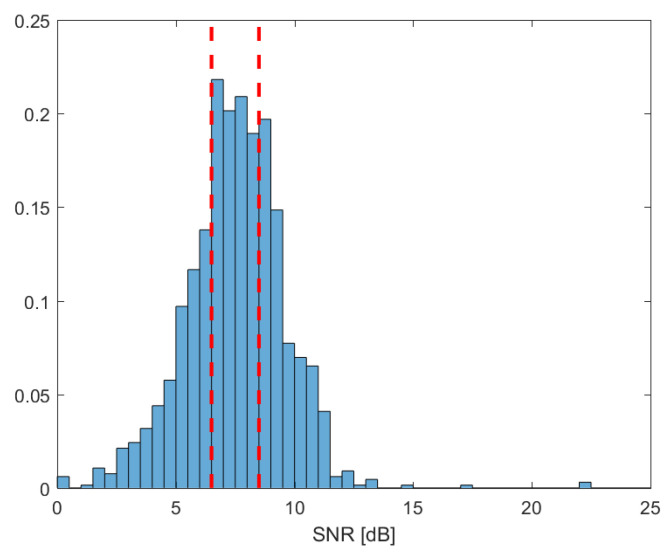
Normalized histogram of the SNRs in dB scale for the utterances in the evaluation set of the CHiME-4 database.

Table 1, Table 2 and Table 3 show the average PESQ score, eSTOI, and SISDR for each method depending on the noise type, respectively. The proposed method with the CDR-based *a priori* SPP estimator, CDR-Proposed, outperformed the previous approach in [26] by 0.39 in terms of the average PESQ score, 0.022 in terms of the eSTOI, and 4.3 dB in terms of the SISDR on average, respectively. With the DNN-based *a priori* SPP estimator, the proposed method, DNN-Proposed, improved the performance of DNN-REMKF by 0.21 in terms of the average PESQ score, 0.017 in terms of the eSTOI, and 1.2 dB in terms of the SISDR on average, respectively. Table 4 shows the PESQ scores, eSTOIs, and SISDRs for the baselines and the proposed method depending on the SNR. As the SNRs for the utterances in the evaluation set of the CHiME-4 database are distributed as in Figure 3, we divided the evaluation set into three groups depending on the SNR: low SNR less than 6.5 dB, medium SNR between 6.5 and 8.5 dB, and high SNR over 8.5 dB. It can be seen that all the measures were improved in all SNR ranges, and the performance improvements were more pronounced in low SNRs. From the results, we may conclude that the proposed speech SCM estimation approach could improve the performance of the multi-microphone speech enhancement method, regardless of the adoption of the DNN for the *a priori* SPP estimation.

### 5.3. Ablation Study

Additionally, we carried out an ablation study to analyze how much each module in the proposed system contributed to the performance improvement. We propose the speech PSD estimator, ${\hat{\phi }}_{s}^{tcs,s}$ in (31), and the RTF estimator, ${\hat{g}}^{tdoa,s}$ in (29). The previous approaches were the speech PSD estimator using recursive smoothing, ${\hat{\phi }}_{s}^{ts}$ in (23), and the ML estimator of the RTF ${\hat{g}}^{ml}$ in (25). The performances of the systems replacing the proposed modules one by one with conventional modules are summarized in Table 5. DNN-REMWF is also included, which uses ${\hat{\phi }}_{s}^{ts}$, ${\hat{g}}^{ml}$, and the Wiener postfilter, but adopts a different noise SCM estimator, derived from the EM framework.

The proposed system with conventional speech PSD and RTF estimators, ${\hat{\phi }}_{s}^{ts}+{\hat{g}}^{ml}$, showed the same average PESQ score and improved eSTOI and SISDR compared with DNN-REMWF [27]. Among the systems in the same framework, the introduction of the proposed speech PSD estimator improved the average PESQ scores by relatively large differences of 0.12 and 0.19, whereas it did not result in increased eSTOIs and SISDRs. On the other hand, employing the proposed RTF estimator improved all three metrics. From the results, we may conclude that both the proposed speech PSD and the RTF estimators contributed to the performance improvement.

### 5.4. Computational Complexity

Additionally, we have compared the computational complexity of the baseline and proposed methods in terms of the normalized processing time for the MATLAB implementation of the methods. The processing times for each algorithm, normalized by the processing time of the proposed algorithm, are given in Table 6. In this experiment, the *a priori* SPP was estimated by a DNN for all cases. As they depend on implementation details and settings such as the number of microphones, sampling frequency, and the dimensions of the DFT, the numbers given in the table should only be used as a rough indication. To see how much the refinement in the second pass incurred additional computational burden, the proposed method without the second pass (denoted as woSP) is included. From the table, it can be seen that the computational complexity of the proposed method was higher than those for MWF [26] and REMWF [27], but less than that of REMKF [27].

## 6. Conclusions

Multi-microphone speech enhancement exploits spatial information and spectro-temporal characteristics to reduce noise from the input. The online algorithms are required for the applications sensitive to time delays such as speech communication and hearing aids. In this paper, we propose an improved estimator of the speech SCM for online multi-microphone speech enhancement. Using the decomposition of the speech SCM under a rank-1 approximation, we propose an improved estimator for the speech PSD and RTF. For speech PSD estimation, we adopt the TCS scheme, which exploits knowledge on the speech signal in the cepstral domain to provide a better estimate of the speech PSD compared with the ML estimate. The RTF is estimated based on the TDoA estimate summarizing the information from all frequency bins. These estimators are evaluated once with input statistics and refined with an estimated clean speech spectrum and power spectrum obtained in the first pass. Our proposed speech enhancement method showed an improved speech enhancement performance in terms of the PESQ score, eSTOI, and SISDR in various noise environments for the CHiME-4 dataset, compared with other online multi-microphone speech enhancement algorithms.

Future work may include the incorporation of other spatial cues such as the inter-channel level differences on top of the inter-channel phase differences [47] into the RTF estimation without resorting to the far-field assumption. We may also investigate a deep learning approach to estimate acoustic parameters such as the speech and noise PSDs and RTF in a causal manner in the MVDR–Wiener filter factorization framework.

## Figures and Tables

**Table 1:** The average PESQ scores for the different algorithms depending on the noise type.

Method	Noise Type				Avg.
	BUS	CAF	PED	STR	
Noisy	1.32	1.24	1.26	1.28	1.27
CDR-MWF [26]	1.93	1.74	1.86	1.74	1.82
CDR-Proposed	**2.35**	**2.15**	**2.18**	**2.17**	**2.21**
DNN-REMWF [27]	2.10	2.01	2.12	2.03	2.07
DNN-REMKF [27]	2.13	2.03	2.15	2.07	2.10
DNN-Proposed	**2.43**	**2.24**	**2.28**	**2.29**	**2.31**

**Table 2:** The average eSTOIs (x100) for the different algorithms depending on the noise type.

Method	Noise Type				Avg.
	BUS	CAF	PED	STR	
Noisy	71.0	66.0	68.8	67.2	68.2
CDR-MWF [26]	83.1	79.7	82.4	80.2	81.4
CDR-Proposed	**85.1**	**83.4**	**83.7**	**82.4**	**83.6**
DNN-REMWF [27]	83.7	82.0	83.9	83.2	83.2
DNN-REMKF [27]	84.3	82.6	84.5	83.9	83.8
DNN-Proposed	**86.0**	**85.3**	**85.9**	**85.0**	**85.5**

**Table 3:** The average SISDRs (in dB) for the different algorithms depending on the noise type.

Method	Noise Type				Avg.
	BUS	CAF	PED	STR	
Noisy	6.79	7.77	8.60	6.85	7.51
CDR-MWF [26]	9.68	9.44	10.69	9.75	9.89
CDR-Proposed	**14.25**	**14.55**	**14.56**	**13.40**	**14.19**
DNN-REMWF [27]	14.40	14.18	14.96	14.64	14.54
DNN-REMKF [27]	14.71	14.42	15.25	14.99	14.84
DNN-Proposed	**15.90**	**16.07**	**16.34**	**15.83**	**16.04**

**Table 4:** The PESQ scores, eSTOIs, and SISDRs for the baselines and the proposed method depending on the SNR.

Method	PESQ Score			eSTOI (×100)			SISDR (in dB)		
	(−*∞*,6.5)	(6.5,8.5)	(8.5,*∞*)	(−*∞*,5)	(6.5,8.5)	(8.5,*∞*)	(−*∞*,6.5)	(6.5,8.5)	(8.5,*∞*)
Noisy	1.22	1.26	1.33	63.3	67.1	74.2	5.04	7.48	9.74
CDR-MWF [26]	1.66	1.83	1.95	76.7	80.7	86.5	8.81	9.80	10.97
CDR-Proposed	**2.07**	**2.22**	**2.33**	**80.6**	**83.2**	**86.9**	**12.35**	**13.95**	**16.14**
DNN-REMWF [27]	1.84	2.07	2.26	79.3	82.8	87.2	12.80	14.41	16.28
DNN-REMKF [27]	1.87	2.10	2.29	80.0	83.4	87.7	13.07	14.70	16.60
DNN-Proposed	**2.15**	**2.32**	**2.44**	**82.7**	**85.2**	**88.5**	**14.38**	**15.83**	**17.78**

**Table 5:** The PESQ scores, eSTOIs, and SISDRs averaged over all noise types for the proposed method with DNN-based *a priori* SPP estimation by replacing the proposed sub-modules with conventional ones, one by one.

Method	PESQ	eSTOI	SISDR
DNN-REMWF [27]	2.07	83.2	14.54
${\hat{\phi }}_{s}^{ts}$ + ${\hat{g}}^{ml}$	2.07	84.5	15.79
${\hat{\phi }}_{s}^{ts}$ + ${\hat{g}}^{tdoa,s}$	2.12	85.4	**16.07**
${\hat{\phi }}_{s}^{tcs,s}$ + ${\hat{g}}^{ml}$	2.19	83.8	15.52
${\hat{\phi }}_{s}^{tcs,s}$ + ${\hat{g}}^{tdoa,s}$ (DNN-Proposed)	**2.31**	**85.5**	16.04

**Table 6:** Comparison of the normalized processing time when the *a priori* SPP was obtained by a DNN.

	MWF [26]	REMWF [27]	REMKF [27]	Proposed (woSP)	Proposed
Process. time	0.614	0.692	1.132	0.704	1.000

## Data Availability

Not applicable.
